# All Together Now: Modeling the Interaction of Neural With Non-neural Systems Using Organoid Models

**DOI:** 10.3389/fnins.2019.00582

**Published:** 2019-06-21

**Authors:** Evelyn Chukwurah, Allison Osmundsen, Shannon W. Davis, Sofia B. Lizarraga

**Affiliations:** ^1^Department of Biological Sciences, University of South Carolina, Columbia, SC, United States; ^2^Center for Childhood Neurotherapeutics, University of South Carolina, Columbia, SC, United States

**Keywords:** pluripotent, organoids, Alzheimer’s, microglia, neuroimmune, neuroendocrine, gut, Zika

## Abstract

The complex development of the human nervous system has been traditionally studied using a combination of animal models, human post-mortem brain tissue, and human genetics studies. However, there has been a lack of experimental human cellular models that would allow for a more precise elucidation of the intricate dynamics of early human brain development. The development of stem cell technologies, both embryonic and induced pluripotent stem cells (iPSCs), has given neuroscientists access to the previously inaccessible early stages of human brain development. In particular, the recent development of three-dimensional culturing methodologies provides a platform to study the differentiation of stem cells in both normal development and disease states in a more *in vivo* like context. Three-dimensional neural models or cerebral organoids possess an innate advantage over two-dimensional neural cultures as they can recapitulate tissue organization and cell type diversity that resemble the developing brain. Brain organoids also provide the exciting opportunity to model the integration of different brain regions *in vitro*. Furthermore, recent advances in the differentiation of non-neuronal tissue from stem cells provides the opportunity to study the interaction between the developing nervous system and other non-neuronal systems that impact neuronal function. In this review, we discuss the potential and limitations of the organoid system to study *in vitro* neurological diseases that arise in the neuroendocrine and the enteric nervous system or from interactions with the immune system.

## Introduction

The formation of the complex nervous system is a fascinating and crucial series of events that occurs during human development. From the formation of the neural plate in the third week of gestation to the migration of neural crest cells throughout the periphery of the embryo, the nascent nervous system undergoes a series of dramatic changes that produce an intricate network of neural cells that make up the central and peripheral nervous systems (CNS and PNS). This is achieved by the careful coordination of multiple cellular events that include: neuronal progenitor cell (NPC) proliferation, neuronal or glial cell differentiation, neuronal morphogenesis, maturation, and migration. After the formation of the different brain regions, neurons establish connections with specific target cells that are critical to their pre-determined functions.

**FIGURE 1:**
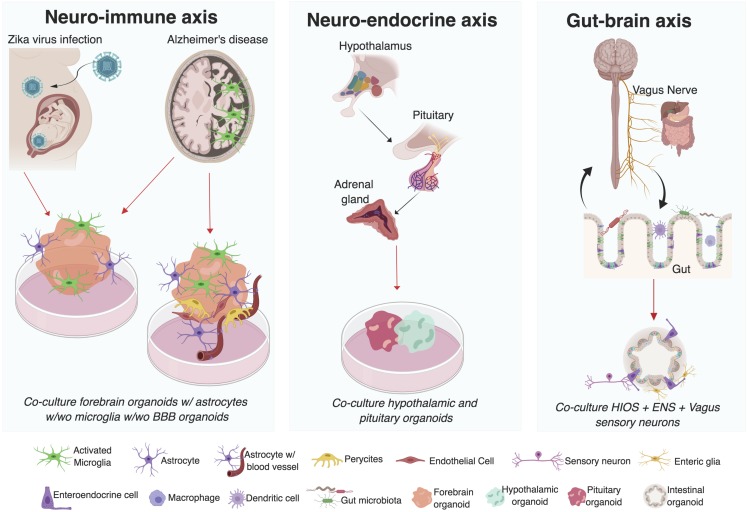
Investigating interactions between multiple systems using organoid co-culture systems. Mechanisms underlying the neuro-immune, neuro-endocrine, and gut-brain axis in humans can be investigated utilizing organoid co-culture systems. Advances have already been made in the use of brain region specific organoids co-cultured with microglia (resident immune cells in the brain) in the study of *ZIKV* induced microcephaly and Alzheimer’s disease. Similarly, advances in the development of blood brain barrier (BBB) stem cell models highlights the exciting possibility of investigating the role of the BBB thinning in AD pathogenesis. Enteric organoids that combine intestinal (HIOs) and enteric nervous system (ENS) cellular components and vagal sensory neurons suggest the possibility to interrogate the mechanisms underlying the gut-brain interactions *in vitro*. Finally, the development of different organoid models of human pituitary and hypothalamic tissue has also been reported. Even though to date there is no organoid model of the adrenal gland, this could potentially be used to interrogate different components of the neuro-endocrine axis. (Figure was created with BioRender.com softwared. Abbreviations used w/wo is with or without).

Most of our current understanding of the intricacies of human nervous system development have come from human genetic studies (Bae et al., 2015), post-mortem tissue analysis (Kretzschmar, 2009; Jhou and Tai, 2017), and work in multiple animal models. Vertebrate and invertebrate animal model systems have given remarkable insight into the genetic programs responsible for the patterning and regionalization of the nervous system that results in different brain structures (Hebert and Fishell, 2008; Doe, 2017). In particular, elegant genetic approaches in murine models have allowed for the systemic or brain region-specific miss-expression of neural genes, leading to the experimental characterization of neural development, and function (Del Pino Rico and Marin, 2018). However, significant differences exist between the murine and human nervous systems, including varying degrees of circuit complexity, and brain architecture (DeFelipe, 2015).  For instance, the lack of gyri and reduced number of outer radial glial cells (oRGCs), a specialized type of neuronal progenitors, in the murine brain compared to the primate brain diminishes the faithful recapitulation of human neurodevelopmental disorders in rodents (Shitamukai et al., 2011; Sun and Hevner, 2014). This highlights the need to establish easily malleable human model systems. This niche was filled in part by the establishment of neuroblastoma cell lines that were amenable to neuronal differentiation and genetic manipulations. Subsequently the establishment of neurosphere cultures from post-mortem fetal brain contributed to the study of neurodevelopmental disorders, such as brain malformations (Sheen et al., 2006; Lu et al., 2011) and Down syndrome (Bahn et al., 2002). Recently, the use of post-mortem human fetal neocortex tissue in combination with single cell transcriptomics (Pollen et al., 2014, 2015; Liu et al., 2016; Nowakowski et al., 2017, 2018) and epigenetic approaches (Yoon et al., 2017a) uncovered temporal and spatial gene programs governing fetal brain development. However, small sample sizes, discreet temporal windows in development, the inability to conduct genetic manipulations, and ethical implications associated with the source of the tissue limit this model system (Farahany et al., 2018). Therefore, the development of human stem cell technology, especially three-dimensional (3D) cultures, constitutes an unparalleled opportunity to study the early stages of human neuronal development in an otherwise inaccessible tissue. These 3D cultures are becoming more complex and have many advantages, such as investigating the interactions between diverse cell types and tissues (Figure 1), but also have limitations, such as the inability to develop a vasculature system, which remain as challenges in the organoid field as it develops (For a summary of advantages and limitations of organoid systems see Table 1; Antoni et al., 2015).

**TABLE 1:** Advantages and disadvantages of organoid systems.

**Advantages**	**Disadvantages**
Access to previously inaccessible tissue that would have only been available post-mortem	Wide variability has been noted in brain organoids cultured under identical conditions
Contain same genetic background as patients in case of iPSC-derived organoids	No standardized protocol for establishing organoid models – Which protocol is the right protocol?
In instances where iPSCs are unavailable, researchers can use genome editing to introduce disease-associated mutations in stem cells to derive organoids	Difficulty in modeling interacting systems requiring organoids to be cultured under different conditions. Trans-well culture systems developed, but prevent direct physical interaction, which may be required
Preponderance of protocols to derive various organoids that can be scaled up or down depending on laboratory capabilities	No established methodology to introduce functional vascularization into brain organoids, short of engrafting the organoids into rodent hosts
Provides a more “*in vivo*” like human cellular model for drug screening	Cultures are more representative of fetal developmental stages rather than postnatal stages
3D microenvironment that more faithfully recapitulates cell-cell interactions than 2D models	Clonal variability, even among iPSCs from the same patient, requires the use of multiple clones to generate organoids

The seminal work by Gurdon (Gurdon et al., 1958; Gurdon, 1960) and the group of Shinya Yamanaka (Takahashi and Yamanaka, 2006; Takahashi et al., 2007) to revert an adult somatic cell to an embryonic state by nuclear reprogramming set the stage for the massive expansion in the use of human induced pluripotent stem cell (iPSC) derived models. Stem cell technology, combined with information on brain patterning signaling pathways, allowed for the development of protocols to induce neuronal (Marchetto et al., 2008; Shi et al., 2012b; Maroof et al., 2013; Kim et al., 2014; Yu et al., 2014; Ahn et al., 2016), and glial cell types (Hu et al., 2009; Shaltouki et al., 2013; Muffat et al., 2016b; Santos et al., 2017) in two-dimensional (2D) *in vitro* cultures. 2D stem cell derived neural models provide a human platform to conduct molecular, cellular, or physiological studies and are amenable to high throughput drug screening (Darville et al., 2016; Rana et al., 2017; Wang C. et al., 2017). These models are uncovering the cellular and molecular mechanisms underlying the pathology of neurological disorders for which animal models fall short, such as Alzheimer’s disease (AD) (Israel et al., 2012; Muratore et al., 2014, 2017; Moore et al., 2015), and schizophrenia (Hook et al., 2014; Hoffman et al., 2017). However, a major limitation of 2D models is that they cannot recapitulate the *in vivo* 3D spatial organization present in the nervous system that results from the interactions of different neural cell types. For example, 2D models can replicate the temporal sequence of events necessary for corticogenesis in the cerebral cortex, but cannot generate its architectural organization, which is critical for understanding the neural networks necessary for normal function and that are disrupted in disease states. Thus, the development of 3D stem cell derived neural model systems has revolutionized how we study the development of the cerebral cortex (Quadrato and Arlotta, 2017; Pasca, 2018b). 3D neural models or brain organoids resemble the multicellular and three-dimensional nature of the structures present in the CNS and can replicate the early stages of brain development (Lancaster et al., 2013; Lancaster and Knoblich, 2014a; Quadrato et al., 2017; Renner et al., 2017). Stem cells grown as embryod bodies embedded in Matrigel, an extracellular matrix material, have been used as the starting material in brain organoid cultures. In the absence of exogenous patterning signals the formation of cerebral organoids composed of multiple brain regions was observed (Lancaster et al., 2013; Quadrato et al., 2017). Recently, by manipulating multiple signaling pathways in these culture systems, researchers have now been able to drive the production of different region-specific brain structures *in vitro* (Jo et al., 2016; Marton and Pasca, 2016; Qian et al., 2016; Birey et al., 2017).

*In vivo*, the generation of neurons from neural stem cells occurs prior to the formation of glial cells. This temporal sequence of neurogenesis and gliogenesis is conserved in 3D culturing systems, as astrocytes were shown to appear much later in culture (Renner et al., 2017). Initially, oligodendrocytes the myelinating cells in the brain, were not observed in self-directed cerebral organoids; however, recent work using a 3D model based on the aggregation of NPCs, showed that after 8 weeks in culture there was evidence of myelination (Pamies et al., 2017). Similarly, the generation of organoids that contain oligodendrocytes, as well as neurons and astrocytes was recently reported (Marton et al., 2019). Furthermore, brain-region organoids lacked microglia, the immune cells in the brain, which come from a non-ectodermal cell lineage. However, protocols that allow for the differentiation of both mesodermal and neural progenitors from iPSC within the same organoid, showed the spontaneous development of phagocytic microglia (Ormel et al., 2018). Another option for generating organoids that mimic *in vivo* conditions of brain development is to establish co-culture conditions that recapitulate the interactions between glial and neuronal cells (Krencik et al., 2017; Sloan et al., 2017; Cvetkovic et al., 2018). For example, organoids that incorporated human microglia were also developed by co-culturing an SV40 human microglia cell line with brain organoids (Abreu et al., 2018). Taken together these different strategies to integrate neural and glial lineages are highly relevant to modeling brain development in a dish, because glial cells are relevant to the formation, and function of neuronal circuitry (Wu et al., 2015; Allen and Lyons, 2018). Organoids have also been employed in the study of the tissue-specific conditions of neuronal and glial function in the PNS as seen in enteric nervous system (McCracken et al., 2011; Fattahi et al., 2016).

Within the context of neurological disease, organoids from patient-derived iPSCs have provided an additional platform to identify key cellular and molecular hallmarks underlying disease pathogenesis (Mariani et al., 2015; Qian et al., 2016; Raja et al., 2016; Bershteyn et al., 2017; Li et al., 2017; Seo et al., 2017). For example Lancaster et al. (2013) reported the first patient derived cerebral organoid model of human microcephaly associated with mutations in the centrosomal gene CDK5RAP2. Similarly, cerebral organoids of Miller-Dieker syndrome patients with a severe form of lissencephaly, showed mitotic defects in oRGCs, which is a critical progenitor for cortical expansion in humans (Bershteyn et al., 2017). Finally, neural organoids derived from patients with idiopathic autism had increased production of inhibitory neurons compared to unaffected controls (Mariani et al., 2015). Therefore, in less than a decade that this technology has been developed it has been key in the study of neurodevelopmental and neuropsychiatric disorders. In the absence of patient-derived iPSCs, CRISPR-mediated gene editing techniques have allowed researchers to introduce disease-associated mutations into stem cells and in so doing, closely capture the neurological disease state (Wang P. et al., 2017) and further highlight the power of the stem cell technology to study human disease.

The proliferation of 3D stem cell derived models provides an expanding scientific playground in which we can begin to ask key questions in human biology using a multi-system approach. During development and in disease, there can be a mutual dependence of the nervous system on other non-neural systems for proper function. A key example is the relationship between the cardiovascular and the nervous system. During embryogenesis the development of the vasculature has been shown to be essential for brain development and function (Paredes et al., 2018). The relevance of the interaction between neuronal and non-neuronal systems is also highlighted by the autonomic and somatic motor systems of the PNS. In these systems, the nervous system regulates the function of non-neuronal tissue by sending and receiving information that could elicit the contraction of skeletal muscle tissue, for example. This prompts the following questions: How well can organoid models recapitulate multi-system interactions? And, can neural circuits that span multiple systems be replicated *in vitro*? Previous reviews on cerebral organoids have focused on the principles of organogenesis and tissue morphogenesis (Lancaster and Knoblich, 2014b; Jabaudon and Lancaster, 2018; Pasca, 2018a), methodologies (Kelava and Lancaster, 2016), integration of different brain regions (Birey et al., 2017; Pasca, 2018a), and the study of development or disease (Muffat et al., 2016a; Quadrato et al., 2016; Amin and Pasca, 2018; Brown et al., 2018; Heide et al., 2018; Pasca, 2018b). Here, we focus on the potential of organoids to model interactions between the nervous system with non-neural systems (Figure 1). We further discuss how alterations in these systems contribute to our understanding of human neuronal development and disease.

## Organoid Modeling of Neuro-Immune and Neuro-Vascular Interactions

Early experiments with dye injections into guinea pig (Wislocki, 1891) and pig embryos (Weed, 1917) showed an astonishing absence of staining in the embryonic brain and spinal cord, suggesting that the CNS was compartmentalized away from the rest of the circulating blood and lymph. Subsequent transplantation of rat sarcoma tissue into mouse skin, muscle, and brain parenchyma showed that shortly after transplantations only the rat tissue transplanted into the CNS was protected from immunological attack (Shirai, 1921). These results suggested that the brain and spinal cord are in a state of *immunological privilege* and are devoid of infectious pathogens present in blood and lymph due to the presence of a blood-brain-barrier (BBB). The BBB is a specialized endothelial cell membrane (Reese and Karnovsky, 1967; Brightman and Reese, 1969) that controls the traffic from the blood into the vascular space of the brain (Banks, 2015). During normal physiological conditions the BBB tightly restricts the passage of immune-reactive molecules and immune cells from the systemic circulation into the CNS (Banks et al., 2012), while allowing passive gaseous exchange and carrier or receptor-mediated transport of select molecules.

A growing body of work suggests that thinning of the BBB allows immune response cells from the circulating periphery to infiltrate the CNS, and in turn induce the chronic neuro-inflammation associated with AD (Regen et al., 2017; Zenaro et al., 2017). These inflammatory conditions are also driven by the activation of the microglia (Regen et al., 2017). Microglia are responsible for the surveillance of the immune landscape in the CNS, arise from hematopoietic precursors that migrate to the neuroepithelium prior to the closure of the neural tube, and differentiate to become the resident CNS phagocytic immune cells (Alliot et al., 1999; Ginhoux et al., 2010; Swinnen et al., 2013). As such, microglia can respond quickly to the presence of pathogens that breach the BBB by exerting phagocytosis (Nicolas-Avila et al., 2018) and acting as traditional antigen presenting cells (Mack et al., 2003). Microglia play key roles in the wiring of the brain by regulating synapse pruning and maturation (Tremblay et al., 2010; Paolicelli et al., 2011; Schafer et al., 2012; Bialas and Stevens, 2013), as well as maintaining brain health by clearing dying or stressed neurons from the CNS (Davalos et al., 2005).

The relevance of neuro-immune interactions in brain health is further highlighted by studies on maternal immune activation in the etiology of neuropsychiatric disorders like autism and schizophrenia (Estes and McAllister, 2016; Nardone and Elliott, 2016; Careaga et al., 2017). In this section, we discuss the use of organoids to model the complex interactions that occur between neurons and glial cells in the neuro-immune axis, especially in the context of Zika virus (*ZIKV*) infections and AD pathogenesis. We also discuss models of the BBB and its role in mediating neuro-immune responses and potentially modeling the neuro-vascular axis.

### Modeling Neuro-Immune Interaction in ZIKV Microcephalic Organoids

The cerebral cortex begins as a single neuroepithelial sheet that borders CSF-filled lateral ventricles. Neuroepithelial progenitor cells are aligned along a basal-apical axis and give rise to specialized proliferative regions. In higher mammals, the developing cerebral cortex contains three proliferative regions: the ventricular zone (VZ), the subventricular zone (SVZ), and the outer subventricular zone (OSVZ) (Lehtinen and Walsh, 2011; Lui et al., 2011). Remarkably this *in vivo* organization of the cerebral cortex is closely mirrored in self-directed and forebrain patterned organoids (Lancaster et al., 2013; Quadrato et al., 2017; Qian et al., 2018), which develop a primitive cortical plate structure and contain neurogenic regions equivalent to those found *in vivo* (VZ, SVZ, and OSVZ). These results suggest that the neurogenic and migratory pathways associated with *in vivo* cortical development are also replicated *in vitro* (Lancaster et al., 2013; Renner et al., 2017; Qian et al., 2018).

By resembling the early fetal brain stages, cerebral organoid technology provides an ideal model to study the early stages of human neuronal development and has been instrumental in the study of *ZIKV* infection of the fetal brain (Song et al., 2017). Expectant mothers who contracted the flavivirus *ZIKV* through the bites of *ZIKV*-infected mosquitoes gave birth to children with pronounced microcephaly (small brain), showing a primarily reduced cerebral cortex (Kikuti et al., 2018). Postmortem studies and 2D neuronal models indicated that NPCs are the primary affected cell types (Mlakar et al., 2016; Tang et al., 2016; Strafela et al., 2017). Several groups have now utilized organoid technology to model infection by *ZIKV* during fetal brain development (Qian et al., 2016; Salick et al., 2017) and to elucidate the molecular underpinnings of *ZIKV* entry into its neural host cell types (Garcez et al., 2016; Wells et al., 2016; Yoon et al., 2017b). These studies showed that ZIKV microcephaly correlates with a decrease in the size of the VZ due to reduced NPC proliferation and increased apoptosis of NPCs (reviewed in Qian et al., 2017; Chen et al., 2019; Setia and Muotri, 2019). Using customized mini-bioreactors and forebrain organoids, Qian et al. (2016) demonstrated that *ZIKV* infection has the most pronounced effect during the first trimester of fetal development, when the developing cortex is predominantly populated by NPCs that will proliferate and differentiate to form the majority of the cerebral cortex.

While the most severe effect of *ZIKV* was seen in NPCs, a remaining question was whether *ZIKV* could utilize glial cells to initiate or perpetuate NPC infection in the developing brain. Muffat et al. (2018) found that while *ZIKV* was able to replicate inside of iPSC-derived astrocytes and microglia, it did not increase cell death in these cell types. Thus, *ZIKV* infected glial cells could serve as putative viral reservoirs within the brain. In particular, *ZIKV* infected microglia could act as Trojan horses to breach the immunological privilege of the brain by bringing in infectious viral loads during their early migration into the neural tube. In fact, NPCs were efficiently infected with supernatant of *ZIKV*-infected microglia (Muffat et al., 2018). Similarly, co-cultures of *ZIKV*-infected microglia with neuralized forebrain cortical organoids showed that microglia migrated into the organoid tissue, matured, and infected organoid NPCs with *ZIKV*, leading to a microcephalic-like phenotype in the organoids (Muffat et al., 2018). Therefore, the infective potential of *ZIKV* in the developing brain might be further amplified by the presence of microglial viral reservoirs. These findings highlight the usefulness of co-culture systems in the study of how different cell types might contribute to the disease phenotype.

*ZIKV* induced microcephaly is an extreme example of how a maternal transmitted infection can have detrimental consequences during fetal brain development. However, it is not the only example, as several studies have shown that inflammation during pregnancy increases the risk for neuropsychiatric disorders, such as autism and schizophrenia (Estes and McAllister, 2016; Careaga et al., 2017). Similarly, the impact of inflammation in the adult brain and neurodegenerative disorders is a growing field of research. Next, we discuss the use of organoids to model the impact of the neuro-immune axis in neurodegeneration.

### Neuroimmune Interactions in the Adult Brain: AD Organoid Models

Alzheimer’s disease affects close to 7% of adults around the world and is characterized by progressive cognitive decline that leads to age-related dementia. The major hallmarks of AD pathology are the accumulation of amyloid-β (Aβ) plaques within extracellular spaces and neurofibrillary tangles, formed by deposition of hyperphosphorylated TAU within neurons. The Aβ hypothesis of AD pathology posits that the accumulation of Aβ plaques leads to neurofibrillary tangles and induces a state of chronic neuroinflammation, which in turn worsens AD pathology (McGeer and McGeer, 2013). In AD, the β- and γ-secretases cleave Amyloid Precursor Protein (APP) to produce larger amounts of a 42 amino-acid long Aβ isoform – Aβ_42_, which is highly prone to aggregating and accumulating in the extracellular space (Dawkins and Small, 2014). The Aβ_42_ aggregates are not efficiently cleared by microglia. However, the microglia do release pro-inflammatory cytokines, which induces a state of neuroinflammation that exacerbates the AD symptoms (Castillo et al., 2017). Furthermore, chronic activation of microglia and increased inflammation has been suggested to exacerbate the activation of kinases that phosphorylate TAU (Asai et al., 2015; Sanchez-Mejias et al., 2016). Hyperphosphorylation of TAU increases microtubule instability, disrupting intracellular trafficking along the microtubules, and contributes to axonal degeneration associated with AD.

Two-dimensional stem cell derived models have been used in studies of familial AD, in which the onset of the disease is linked to mutations in one of three genes: APP, which leads to increased APP production, or Presenilin 1 and 2 (PSEN1 and PSEN2), which results in diminished APP processing (Israel et al., 2012; Shi et al., 2012a; Muratore et al., 2014). These studies showed that AD patient-derived neurons had increased pathogenic Aβ peptide production and increased TAU phosphorylation (Israel et al., 2012; Muratore et al., 2014; Saurat et al., 2016) that depended on the length of time in culture (over 100 days in culture) (Muratore et al., 2014; Saurat et al., 2016). However, these cultures did not recapitulate the accumulation of amyloid plaques or intracellular neurofibrillary tangles that are hallmarks of AD. Subsequent attempts to generate a suitable microenvironment in which secreted Aβ peptide could lead to the formation of amyloid plaques and intracellular neurofibrillary tangles were made using a 3D stem cell derived neural model overexpressing AD associated mutations in APP and PSEN1 (Choi et al., 2014). Similarly, AD cellular phenotypes were observed in PSEN1 patient derived self-organizing cerebral organoids cultured for over a 100 days in a spinning bioreactor (Lancaster et al., 2013; Kim et al., 2015; Gonzalez et al., 2018), as well as in iPSC-derived neural organoids with APP duplications (*APP ^Dpp^1-1* and *APP ^Dpp^2-3*) and PSEN1 mutation (*PSEN1^A264E^*) (Raja et al., 2016). However, these models did not address the role of inflammation in the progression of AD, as these organoids did not account for the role of astrocytes or microglia in their observed phenotypes. The fact that postmortem studies showed increased microglia and activated astrocytes surrounding Aβ plaques in AD patients compared to control samples (Vehmas et al., 2003), emphasizes the importance of incorporating microglia and astrocytes into brain organoid models of AD.

Forebrain organoid models that are fated to a neuroectodermal fate produce multiple neuronal cell types, but require long-term cultures to generate astrocytes, and usually do not produce microglia, which is of mesodermal origin (Pasca et al., 2015; Sloan et al., 2017). Alternatively, self-directed organoid models generated in the absence of patterning molecules showed the presence of mesodermal lineage progenitors leading to the spontaneous development of phagocytic microglia (Ormel et al., 2018). Thus, self-directed organoids could be used to investigate the role of microglia in neurological disorders and neurodevelopment. Furthermore, the development of protocols to robustly generate microglia from human stem cells makes it possible to assess the role of microglia in AD pathogenesis. This system was used to investigate the pathogenicity of Apolipoprotein E (APOE) protein alleles in AD. APOE is a cholesterol transporter that has been shown to be associated with elevated risk for developing sporadic AD (Lopez et al., 2014). APOE is primarily expressed by astrocytes and to a lesser extent by microglia and neurons, and was found to colocalize with neurofibrillary tangles and Aβ plaques in postmortem AD tissue, demonstrating a possible connection to AD pathogenesis (Namba et al., 1991). In humans, the *APOE* gene has three polymorphic alleles: *APOE2, APOE3*, and *APOE4*, each with different binding affinities for Aβ plaques (Hashimoto et al., 2012). Analysis of post-mortem brain tissue from AD-afflicted individuals showed a strong correlation between the presence of *APOE4* allele and the severity of AD hallmark pathologies. Similarly, embryonic stem cell derived neurons carrying the *APOE4* allele showed higher production of Aβ peptide compared to the more common allele *APOE3* (Huang et al., 2017). CRISPR/CAS9 genome editing was used to investigate *APOE4* cell specific AD pathogenesis in isogenic iPSC lines were the *APOE4* allele was introduced into a non-AD patient *APOE3* iPSC lines (Lin et al., 2018). To determine the impact of microglia in AD pathology iPSC-derived *APOE3* or *APOE4* microglia were co-cultured with APP^Dpp^ (APP duplication) forebrain cortical organoids. *APOE4* did not affect the migration or incorporation of microglia into forebrain organoids. However, it induced morphological changes in the organoid neurons, such as elongated processes with increased Aβ fragments. These results suggest that *APOE4* microglia might be defective in clearing the Aβ peptides (Lin et al., 2018).

While the role of microglia in AD is becoming well established, the role of astrocytes in AD pathogenesis remains poorly understood. Astrocytes produce their own inflammatory molecules in response to cytokines and may contribute to the pro-inflammatory response in the CNS of familial or sporadic AD patients (Rothhammer and Quintana, 2015). Already analysis of iPSC-derived *APOE4* astrocytes showed reduced clearance activity of Aβ peptides compared to isogenic control astrocytes (Lin et al., 2018). However, the additive effect of AD mediated inflammation on astrocytes could not be tested in this 2D neural model. The development of long-term cerebral organoid cultures that generate astrocytes (Sloan et al., 2017) will provide an elegant model in which to assess the contribution of astrocytes to familial or sporadic AD. In summary, 3D neural models that integrate neuronal and glial cells are allowing us to investigate additional compounding factors that exacerbate AD pathogenesis, like inflammation.

### Perspectives on Modeling the BBB: Implications on the Neuro-Immune and Neuro-Vascular Axes

In the 3D models of AD and *ZIKV* infection discussed thus far, the role of the BBB in establishing the pathological conditions was not examined. With respect to AD, a major remaining question is how do activated circulating immune cells breach the BBB to stimulate astrocytic and microglial activation and promote inflammatory conditions conductive to disease progression? With respect to *ZIKV* induced microcephaly, a major remaining question is what cell types in the BBB are susceptible to *ZIKV* viral infection, and what receptors are expressed in these cells that ease viral entry into *ZIKV*-target cells in the CNS? The development of stem cell derived BBB multicellular models will present a major step forward in unraveling human specific phenotypes associated not only with the impact of disease in the BBB (Lippmann et al., 2014; Lauschke et al., 2017; Campisi et al., 2018), but will also further our current understanding on how the neurovasculature contributes to neuronal development.

Different BBB models have been reported that contain cell types that constitute the BBB and recapitulate in part the function of the BBB. Two models using human primary astrocytes, vascular pericytes, and brain microvascular endothelial cells or immortalized cerebral endothelial cells have been described (Cho et al., 2017; Bergmann et al., 2018). In addition, a cortical spheroid model with a functional BBB was generated utilizing a hanging drop method that mixed in six cell types present in the BBB (endothelial cells, pericytes, astrocytes, microglia, oligodendrocytes, and neurons) (Nzou et al., 2018). Although this model did not survive long in culture, it did show the formation of spheroids that were selectively permeable to various compounds. While this spheroid model is not a proper brain organoid it highlights the potential of future co-cultures that incorporate brain organoids with microglia, astrocytes, and BBB organoids. In fact, recent work from Song et al. (2019) demonstrated that the fusion of cortical neuronal progenitor spheroids with endothelial spheroids and mesenchymal stem cells lead to the formation of a hybrid neurovascular organoid that develops a structure akin to a rudimentary BBB that expressed markers for tight junctions, like ZO1, which are hallmarks of endothelial barrier tissue. While these models allow us to study the role of the neurovasculature in development and disease they are still at their infancy and further research is needed to identify the specific cell types within the BBB necessary for viral entry and the cognate receptors necessary for pro-inflammatory cytokine entry into the developing fetal CNS. These experiments can be facilitated using organoids developed from receptor-depleted iPSC-derived BBB cells (Lippmann et al., 2013). Finally, while a major challenge to the brain organoid models has been the inclusion of vasculature, strategies creating these hybrid neurovascular organoids could potentially surpass this challenge. In addition, the incorporation of bioengineering technology into brain organoid research, as has been done on kidney organoids that were cultured under flow on millifluidic chips and resulted in the generation of a vascular network with lumens, could potentially contribute to more “*in vivo*” like neurovascular organoid models (Homan et al., 2019).

## Gut Feeling: Modeling the Enteric Nervous System in 3D

There is extensive cross talk between the PNS and the immune system, as evidenced by the extensive innervation of lymphoid organs by sympathetic post-ganglionic nerves as well as by the vagus nerve (Felten, 1993; Zila et al., 2017). Furthermore, several immune response cells release neuropeptides in response to infection and express receptors for various neurotransmitters (Felten et al., 1993). Activation of receptors by neurotransmitters stimulates the proliferation and maturation of the immune response cells promoting a timely response to pathogenic presence in the PNS (Pinho-Ribeiro et al., 2017). This could highlight a potential connection between inflammation and gastrointestinal impairments observed in neurodegenerative and neuropsychiatric disorders. In addition to AD, Parkinson’s disease has a clear presentation of gastrointestinal (GI) impairments (Su et al., 2017). Similarly, GI problems are also associated with autism (Dalton et al., 2014) and have been reported in syndromic autism such as Christianson syndrome patients (Pescosolido et al., 2014). However, the extent to which GI impairments contribute to neurological pathologies in each of these disorders is not well understood. In this respect an emerging field is the study of how the gut microbiome might impact neurodevelopmental and neuropsychiatric diseases. This topic has been extensively reviewed (Fung et al., 2017; Bruce-Keller et al., 2018) and is not revised here. Instead, we focus on 3D models that integrate the enteric nervous system (ENS) or the “*brain in the gut*” and GI tissue. The ENS is the largest sensory organ in the body and is formed by a network of neural cells resident in the GI tract.

### The Complex Development and Function of the ENS

Upon closure of the neural tube, neural crest cells migrate into the primitive gut to form the future ENS. The ENS is part of the autonomic nervous system and modulates peristaltic reflexes, fluid secretion into the lumen of the GI tract, and vasodilation of GI tract blood vessels (Kiba, 2006). The ENS is an extensive network of neurons that innervate the entire GI tract and works in conjunction with non-neuronal enteroendocrine cells and interstitial cells of Cajal (ICC) to provide reflex functions that are somewhat autonomous from the CNS (Rao and Gershon, 2018). Enteroendocrine cells secrete a wide array of neurotransmitters and signaling molecules to stimulate enteric neuronal activity in response to the changing GI environment. ICCs are the resident intestinal pacemaker cells and modulate smooth muscle contraction independent of neuronal input. The regulation of the peristaltic reflex by the ENS is triggered by the activation of intrinsic primary afferent neurons (IPANs) by GI luminal distension or neurotransmitters such as serotonin, which are released by the enteroendocrine cells. IPANs, project their axons into the intestinal mucosa, transmitting their signals to ascending and descending interneurons. Ascending interneurons synapse with cholinergic (ChAT^+^) excitatory motor neurons eliciting contraction of the intestinal smooth muscle proximal to the site of IPAN activation by acetylcholine (Ach) release. Descending interneurons synapse with inhibitory motor neurons and induce smooth muscle relaxation distal to the site of IPAN stimulation by release of multiple neurotransmitters including: nitric oxide and vasoactive intestinal peptide (VIP). The combination of contraction and relaxation of the intestinal smooth muscles is the basis for the peristaltic reflex in the GI tract. Glial cells are also present in the ENS, and like the astrocytes in the CNS, protect the endothelial barriers in the GI while also maintaining optimal conditions for neuronal function. However, the cellular complexity of the ENS makes it challenging to model *in vitro*.

### The Evolving Landscape of Gastrointestinal and ENS Organoids

Waardenburg syndrome and Hirschsprung’s disease are severe enteric neuropathies that are often fatal; thus, highlighting the essential role of ENS in regulating intestinal tissue activity (Brosens et al., 2016). Unfortunately, studying the molecular underpinnings of these neuropathies has been nearly impossible due to a dearth of ENS models with the spatial complexity of the human intestine. Recently, at least two strategies have been developed to derive human intestinal organoids (HIOs) that incorporate ENS like tissue (Schlieve et al., 2017; Workman et al., 2017). A combination of TGF-β, WNT3A, and FGF4 signaling was used to induce embryonic stem cells (ESCs) or iPSCs to generate mid and hindgut tissues. These HIOs had similar epithelial cell and mesenchymal distribution as normal human intestinal tissue, but lacked ENS cells (McCracken et al., 2011; Spence et al., 2011). Vagal neural crest progenitor cells (NCCs) derived from iPSCs by using retinoic acid (RA) were subsequently incorporated into HIOs (Fattahi et al., 2016; Workman et al., 2017). Immunohistochemical staining of HIOs aggregated with NCCs (termed HIOs + ENS organoids) revealed that enteric neurons and glia populated the smooth muscle layers of the HIOs, like the enteric ganglia do *in vivo* in the GI tract. However, they did not contain all the enteric neural cell types as only IPANs, ChAT^+^ excitatory neurons, dopaminergic neurons, and some types of inhibitory neurons were observed. Notably HIOs + ENS lacked glia as well as enteric inhibitory neurons, which actively express nitric oxide synthase (NOS) and function in smooth muscle relaxation. The functionality of *in vitro* derived organoids in terms of their capacity to mature and integrate *in vivo* has been tested in “chimeric” models transplanting human stem cell derived tissue into live animals. HIOs + ENS engrafted into live mouse kidneys had increased NOS-expressing inhibitory neurons, reduced ChAT^+^ neurons, and diminished numbers of enteric neuronal cell bodies compared to actual human intestine. These results suggest that aggregation of NCCs with HIOs do not contain all of the intrinsic cues that are essential for the timely appearance of ENS excitatory and inhibitory neurons. Alternatively, the kidney environment might not have been as supportive for ENS development as would have been engraftment into the GI. Despite the alterations in neuronal cell type composition, neurons within the HIOs + ENS were functional as shown by Ca^2+^ efflux measurement. HIOs + ENS neurons mediated sustained intestinal smooth muscle contraction and relaxation independent of ICCs. An important discovery using the HIOs + ENS model is that the ENS promotes cellular proliferation and differentiation of intestinal cells. HIOs + ENS, but not HIOs alone, lead to increased cell proliferation in the intestinal crypt, which correlated with increased expression of genes regulating proliferation and intestinal cell development.

Schlieve et al. (2017) reported the development of a more *“in vivo”* like ENS containing organoids enriched in multiple enteric neuronal and glial cells. ENS neurons were derived from iPSCs cultured in RA, inhibitors of BMP and TGF-β signaling, and activators of GSK signaling. A key difference with the HIOS + ENS method, was that NCCs and HIOs were seeded onto polyglycolic acid scaffolds, and were implanted into the omentum of irradiated mice for *in vivo* maturation. The omentum is a fold of the peritoneum connecting the stomach with other abdominal tissues. Therefore, this could provide a more permissive environment to the development of more *in vivo* like ENS. This organoid model referred to as ENCC-HIO-TESI (enteric neural crest cells – human intestinal organoid – tissue engineered small intestine) contained epithelial and mesenchymal cells associated with mature human intestinal tissue. ENCC-HIO-TESI also contained excitatory ChAT^+^ neurons, serotonin^+^ IPANs, ascending and descending interneurons, inhibitory NOS^+^ neurons and enteric glial cells. Enteric glia (SOX10/GFAP^+^ and SOX10/S100β^+^) was also found throughout the ENCC-HIO-TESI mesenchyme. This was in stark contrast to the HIOs + ENS (Workman et al., 2017), where excitatory ChAT^+^ and inhibitory NOS^+^ neurons where not present simultaneously and lacked enteric glia. While HIOs + ENS had an apparent lack of neuroepithelial connections, confocal microscopy analysis of ENCC-HIO-TESI showed that axons from enteric neurons projected into the cell bodies of enteroendocrine cells (Schlieve et al., 2017). Similar to HIOs + ENS, neurons in ENCC-HIO-TESI were also shown to regulate contraction and relaxation of intestinal muscles. Therefore, ENCC-HIO-TESI restored connections between enteric neural and non-neural cells and could more faithfully recapitulate the mutual dependence of each cell type in ensuring proper intestine function.

A future challenge in the study of the GI and ENS *in vitro* will be the development of *in vitro* culturing conditions that mimic the omentum environment. The combination of NCC and HIOs derived from separate populations of stem cells demonstrates the success and potential of co-culturing organoids for modeling human tissues. The development of organoid cultures that contain multiple progenitor populations is also observed in the development of organoid cultures in the neuroendocrine axis.

## Organoid Modeling of the Neuroendocrine Axis

### Pituitary Development *in vivo*

The endocrine system refers to a collection of glands that secrete hormones to regulate various physiological processes, like metabolism, reproduction, and growth. The pituitary gland is often nicknamed the “master gland” because its hormones help regulate other endocrine glands, including the thyroid gland and adrenal glands. Mutations in genes that control pituitary development may result in hypopituitarism, or a deficiency in a single or multiple pituitary hormone cell types. On the other hand, pituitary adenomas can cause the secretion of excess hormones, leading to disorders such as gigantism, or Cushing’s disease. Current treatment for pituitary dysfunction includes hormone replacement therapy. However, not all pituitary hormones have replacement therapy, some therapies are typically discontinued after puberty, and proper dosage is complicated and variable. For example, it is difficult to properly mimic the circadian or stress-induced change in hormone levels (Suga et al., 2016). Therefore, pituitary cell replacement therapies would greatly improve upon current hormone replacement treatments.

The pituitary gland is derived from two different ectodermal structures; namely the oral and neural ectoderm. Oral ectoderm will produce Rathke’s pouch, the precursor for the pituitary anterior lobe. Neural ectoderm will give rise to the pituitary posterior lobe via an infundibulum of the ventral diencephalon. The patterning of the ventral diencephalon, which includes an organizing center expressing BMP4, FGF8, and FGF10, is important both for the formation of the posterior lobe itself, and for generating the pituitary progenitors in Rathke’s pouch (Davis et al., 2013). Canonical WNT signaling in the ventral diencephalon promotes the expression of this pituitary organizing center, especially the expression of FGF8, which controls the proper size and shape of Rathke’s pouch (Osmundsen et al., 2017).

After the formation of the pituitary gland, the endocrine system and nervous system continue to be intimately linked through hormone secretion. The hypothalamus, which develops from the ventral portion of the diencephalon serves to integrate sensory inputs, process them, and effect responses to regulate basic needs such as stress responses, energy and fluid balance, growth, and even emotional homeostasis. This is accomplished through the release of hormones from the axon terminals of the neuroendocrine neurons into the fenestrated portal blood capillaries of the median eminence and pituitary posterior lobe (Pearson and Placzek, 2013). The proximity of these axon terminals and portal vessels allows hypothalamic neurohormones and neurotransmitters to be released into the bloodstream for delivery to target cells. In the posterior lobe, pituicytes, which are astrocytic-like cells, can modulate the release of neurohormones (Wittkowski, 1998). In the median eminence, radial glial-like tanycytes are thought to support the bidirectional flow of components between the hypothalamus and circulatory systems (Wittkowski, 1998). Regulating peptides, including growth hormone-releasing hormone (GHRH), somatostatin (SST), dopamine (DA), thyroid-releasing hormone (TRH), and corticotropin-releasing hormone (CRH), can also be released and passed through the pituitary portal system to control the production and release of anterior pituitary hormones (Pearson and Placzek, 2013).

### Neuroendocrine Deficits in Neurodevelopmental Disorders

Prader-Willi syndrome (PWS), a complex multisystem genetic disorder, is characterized by short stature, muscle hypotonia, hyperphagia, obesity, hypogonadism, behavioral problems, and developmental delay (Holm et al., 1993; Goldstone et al., 2008; Angulo et al., 2015). Children with PWS have impaired growth, most likely caused by a combination of growth hormone (GH)/insulin-like growth factor 1 (IGF-1) deficiency and a lack of pubertal growth spurt (Driscoll et al., 1993; Grugni et al., 2011; Bakker et al., 2017). Most PWS patients fall into the mildly intellectually disabled range, have multiple severe learning disabilities, and poor academic performance (Whittington et al., 2004). In addition, children with PWS are at risk for autism spectrum disorder (ASD), including pervasive social deficits (Zyga et al., 2015). To treat PWS, individuals are given GH therapy (Aycan and Bas, 2014). However, reports of sudden death have occurred in PWS patients on GH, and those undergoing treatment should exercise extreme caution (Van Vliet et al., 2004; Bakker et al., 2007). In addition to PWS, there have been multiple reports of other chromosomal abnormalities that bridge endocrine defects and neurodevelopmental disorders. For example, individuals with Tetrasomy X, a rare aneuploidy seen in girls, have facial dysmorphism, premature ovarian insufficiency, intellectual disability, and according to some reports, skeletal abnormalities (Uppal et al., 2017). Additional, patients with Tetrasomy X developed early growth hormone deficiency and later developed partial secondary adrenal insufficiency and central hypothyroidism (Uppal et al., 2017). There has also been a report of a male subject with a maternally inherited deletion of approximately 5.8 Mb at Xq21.1, who has shown developmental delay, dysmorphic facial features, cleft palate, intellectual disability, short stature, and hearing loss, which correlated with multiple pituitary hormone deficiency (Giordano et al., 2015). Another patient with Xq21.1 deletion was found to have severe growth retardation, moderate mental retardation, microcephaly, cleft palate, hearing loss, and secondary adrenal insufficiency due to isolated ACTH deficiency (Kajantie et al., 2004). Taken together, a better understanding of the interactions between the endocrine system and the nervous system may allow for better diagnosis or treatment of these disorders (Figure 1).

### Recapitulating Pituitary and Hypothalamus Organogenesis *in vitro*

Since 3D organoids can be used to model embryonic development, they are ideal for studying the development of the pituitary gland and hypothalamic tissue. Watanabe et al. (2005) and Eiraku et al. (2008) established a three-dimensional suspension culture method for ES cells called “serum-free culture of embryoid body-like aggregates with quick re-aggregation (SFEBq).” This culture method utilizes aggregates of ES cells in culturing conditions suitable for differentiation into ectodermal derivatives. Under these conditions, the ES cells also spontaneously form highly ordered structures and display self-organization (Sasai et al., 2012). Using SFEBq cultures, Wataya et al. (2008) was able to induce hypothalamic neurons from mouse ES cells. The hypothalamic neurons were most efficiently produced in growth factor-free, chemically defined medium (gfCDM) and in the presence of Sonic Hedgehog (SHH), which lead to the differentiation of rostral-ventral hypothalamic precursors and neurons (Suga et al., 2016). Removing exogenous growth factors, instead of adding inductive signals, played a key role in rostral hypothalamic specification and suggests that the default fate of mouse ES cells is rostral hypothalamus (Wataya et al., 2008).

In addition to establishing hypothalamic tissue from mouse ES cells using the SFEBq cultures, researchers were also able to generate pituitary hormone cell types. By increasing the size of the ES cell aggregate Suga et al. (2011) established culture conditions that allowed oral ectoderm to co-exist with hypothalamic tissue. Ochiai et al. (2015) also showed that differentiation into non-neural ectoderm was facilitated by low concentrations of exogenous BMP4 treatment. Since SHH is essential for providing midline identity in the ventral diencephalon (Zhu et al., 2007), Suga et al. (2011) added the smoothened agonist (SAG), which stimulates the SHH signaling pathway. With this addition, ES cell aggregates formed vesicles that expressed LHX3, a marker for the pituitary progenitors in Rathke’s pouch. These pituitary progenitors were able to differentiate into all of the anterior lobe hormone cell types, with adrenocorticotropic hormone (ACTH) secreting corticotropes being the most efficiently produced (Suga et al., 2016). Suga et al. (2011) then transplanted these ACTH secreting pituitary organoids into the kidney capsule of a hypophysectomized mouse. After 1 week, blood ACTH levels were slightly, but significantly increased, and corticosterone, which is released from the adrenal gland upon ACTH stimulation, was also significantly increased. This suggests that the pituitary organoid was able to respond to endogenous signals and restore the hypothalamic-pituitary-adrenal axis, despite its ectopic location in the kidney capsule (Suga et al., 2016). These results demonstrate the feasibility of cell replacement therapy to treat hypopituitarism.

Ozone et al. (2016) were able to recapitulate pituitary development *in vitro* in human ES cells using the SFEBq method. They found that the anterior pituitary self-formed after these large aggregates generated both hypothalamic and oral ectoderm. Interestingly, a single aggregate could contain multiple LHX3+ pouches, while only one pouch normally develops in the embryo. Unfortunately, there did not seem to be a high success rate for the development of these pouches *in vitro*, with only 4.0 ± 0.4 vesicles per eight aggregates, suggesting that the organoid culture conditions can be improved to more efficiently generate pituitary progenitors. The most common cells types produced in the hESCs pituitary organoids are corticotrophs and somatotrophs (Ozone et al., 2016). Gonadotropes and thyrotropes are not efficiently produced in these cultures, suggesting that additional work is needed to generate these cell types in the quantities needed for cell replacement therapy.

The key interaction in the large aggregates in SFEBq culture is likely the interaction between neural ectoderm and surface ectoderm, which can generate a placode, similar to the adenohypophyseal placode that generates Rathke’s pouch. Zimmer et al. (2016) used human pluripotent stem cells (hPSCs) to generate pituitary hormone cell types in 2D culture by first inducing cranial placode progenitors from the hPSCs and then driving the differentiation of pituitary progenitors and specific hormone cell types by manipulating key signaling pathways in culture. Their data indicates that pituitary cell fate can be induced independent of the self-organization seen in the SFEBq 3D culture (Zimmer et al., 2016). ACTH secreting cells generated in this manner were transplanted subcutaneously into hypophysectomized rats and displayed evidence of survival and hormone release for up to 7 weeks after transplantation. This process of transplantation of individual pituitary cells is a promising route for the restoration of the hypothalamic-pituitary axis function in patients with a deficit (Rizzoti et al., 2016). However, patients with combined pituitary hormone deficiency will either require cell replacement with multiple cells types generated separately or the transplantation of a pituitary organoid containing multiple anterior pituitary hormone cell types. The pituitary gland is a highly organized 3D structure where each cell type is part of an interconnected network that responds to hypothalamic releasing hormones in an organized fashion (Le Tisser et al., 2012). While pituitary hormone cell types can be generated and studied in 2D culture, the ultimate goal for cell replacement therapy should be the generation of pituitary organoids that mimic the 3D structure of the pituitary gland.

## Challenges and Future Perspectives

Formation of forebrain, retinal tissue, choroid plexus and hindbrain were original observed by Lancaster et al. (2013) in stem cell derived “mini-brains” that were generated in the absence of exogenous patterning signals (Muratore et al., 2014). While exciting, one drawback of this system is that it is highly variable. Even among organoids cultured simultaneously under the same conditions in different vessels there is heterogeneity in terms of the cell type diversity (Quadrato et al., 2017). Development of brain-region specific organoids by addition of different morphogenetic proteins that provide specific patterning cues has increased the reproducibility of the system (Pasca, 2018b). This approach allows us to interrogate brain-region specific questions, but it limits us in terms of the study of neuronal connectivity. To address this issue, the integration of individually generated dorsal and ventral cortical brain regions was able to generate inhibitory interneurons that recapitulated their *in vivo* tangential migration (Birey et al., 2017) and the assembly of rudimentary neural circuits. This is a promising start, but a major challenge is generating organoids that display the complexity of mature human neural circuits. Part of this challenge is being met by transplantation experiments in mice in which human brain organoid grafts form functional neuronal networks with their host (Mansour et al., 2018).

As organoids grow in size and complexity, another major challenge is to provide oxygenation, nutrients, and structural integration of the vasculature to the organoids. Current methodologies have allowed researchers to grow brain organoids up to 510 days (Sloan et al., 2017, 2018) in the absence of a vascular system. However, the vascular niche is essential for proper brain development and function (Paredes et al., 2018), and efforts are being made to develop culturing conditions that incorporate a vascular system. Transplantation of human cerebral organoids into the murine brain showed the presence of blood vessels in the grafts (Mansour et al., 2018). Similarly, another report showed incorporation of endothelial cells into brain organoids *in vitro*, which were only shown to mature into blood vessels after transplantation into a live animal. iPSC-derived cerebral organoids were co-cultured for up to 3 to 5 weeks *in vitro* with endothelial cells from the same donor and transplanted at day 54 and analyzed after 2 weeks (Pham et al., 2018). The formation of mature blood vessels only after transplant suggests that co-culturing of brain organoids with endothelial cells alone is not enough to allow the maturation of the vasculature *in vitro*. Future directions might incorporate bioengineering approaches like 3D printing of blood vessels or “organ in a chip” type of technology (Lin et al., 2019) to overcome the challenge of vascularization of brain organoids in a complete *in vitro* system.

With respect to the neuro-immune axis one major challenge will be tomodel how neuroinflammation might affect fetal development. Growing evidence suggests a role for maternal inflammation during fetal development as a contributing factor to the complex etiology of neuropsychiatric disorders like autism and schizophrenia (Labouesse et al., 2015; Di Marco et al., 2016). Currently, animal models and epidemiological studies provide the majority of the evidence that suggests that maternal inflammation is correlated with neuropsychiatric disorders. To directly address this hypothesis in human tissue, co-culture systems that incorporate microglia and astrocytes with region specific brain organoids could be expanded to incorporate different brain regions. This “assembloyd” system could be used to test the cell autonomous effect of disease specific mutations in the formation of neural networks *in vitro*. Alternatively, the impact of different immune signaling molecules could also be assessed in co-culture systems of microglia and brain specific organoids, in self directed cerebral organoid systems that contain microglia or in “assembloyds.”

With respect to the Gut-brain axis the recent key discovery that a subset of ENS enteroendocrine cells can directly synapse with cranial nerve axon terminals (Kaelberer et al., 2018) opens up the possibility that GI environmental cues (i.e., microbiota) could directly communicate with the CNS. Kaelberer et al. (2018) examined this circuit *in vivo* using transsynaptic spread of a GFP labeled rabies virus followed by electrophysiological measurements in response to glucose uptake. This circuit was recreated *in vitro* using a co-culture system of enteroendocrine cells and nodose vagal neurons (Kaelberer et al., 2018). Future studies that incorporate patient iPSC-derived GI and ENS organoid models with vagal nerve neurons could provide additional mechanistic insights into the gut-brain connection in disorders such as Parkinson’s disease, which has a known GI involvement.

Finally, with respect to the neuroendocrine axis, the assembly of multiple components this system could interrogate mechanisms underlying neural interactions with the hypothalamic-pituitary-adrenal gland axis, which has been previously associated to individuals at high risk of developing psychosis. While at the moment there are organoid models of adrenal glands, one could envision that being able to develop the hypothalamic-pituitary-adrenal gland axis in a dish could provide mechanistic insight on how corticosteroids could modulate neural circuits in the hypothalamus.

## Author Contributions

SBL, EC, and AO conceived and developed the manuscript. SBL, EC, AO, and SD wrote and edited the manuscript.

## Conflict of Interest Statement

The authors declare that the research was conducted in the absence of any commercial or financial relationships that could be construed as a potential conflict of interest.
